# Supplementary material for: An embryo of protocells: The capsule of graphene with selective ion channels
Source: Sci Rep. 2015 May 19;5:10258. doi: 10.1038/srep10258 (PMC4437305; doi:10.1038/srep10258)
Supplement: Supporting Information [file srep10258-s1.pdf]

Supplementary Information

An embryo of protocells: The capsule of graphene with selective ion channels

Zhan Li [1,2,†], Chunmei Wang[3,†], Longlong Tian[4,†], Jing Bai[2], Huijun Yao[2,*],Yang Zhao[5], Xin Zhang[2], Shiwei Cao[2], Wei Qi[4], Suomin Wang[7], Keliang Shi[4], Youwen Xu[8], Zhang Mingliang[1], Bo Liu[4], Hongdeng Qiu[1*], Jie Liu[4], Wangsuo Wu[4,*], Xiaoli Wang[3], An Wenzhen[9]

1, Key Laboratory of Chemistry of Northwestern Plant Resources, Key Laboratory for Natural Medicine of Gansu Province, Lanzhou Institute of Chemical Physics, Chinese Academy of Sciences, Lanzhou, 730000, P.R. China

2, Institute of Modern Physics, Chinese Academy of Sciences, Lanzhou, 730000, P.R. China

3, Lanzhou Institute of Husbandry and Pharmaceutical Sciences of CAAS, Lanzhou, 730000, P.R. China

4, Radiochemistry Laboratory, Lanzhou University, Lanzhou, 730000, P.R. China

5, Department of Chemistry, State Key Lab of Molecular Engineering of Polymers, Shanghai Key Lab of Molecular Catalysis and Innovative Materials, and Collaborative Innovation Center of Chemistry for Energy Materials, Fudan University, Shanghai 200433, P. R. China.

6, School of Nuclear Science and Technology, Lanzhou University, Lanzhou, 730000, P.R. China

7, State Key Laboratory of Grassland Agro-ecosystems, College of Pastoral Agriculture Science and Technology, Lanzhou University, Lanzhou 730000, P.R. China

8, Brookhaven National Laboratory Medical Department, Building 901, Room 106, Upton, NY 11973

9, School of life science, Lanzhou University, Lanzhou, 730000, P.R. China

[†]These authors contributed equally to this work.

[*]Correspondence should be addressed to: yaohuijun@impcas.ac.cn; wuws@lzu.edu.cn.

External Databases

Formation mechanism of CGOs

GOs contain high levels of sp2 carbons, such that the oxygen level is low. To generate a high concentration of sp3 carbon in GOs, the oxygen level must be increased. Therefore, the graphite was oxidized for different durations (from 8 to 70 h) to prepare GOs HOGOs with high oxygen levels through an improved Hummers method [1]. GO and HOGOs characterization experiments were performed through TEM, Raman spectroscopy, FTIR, Potentiometric titration curve, and XPS (Figs. S1-2). The results showed that HOGOs with more sp3 of carbons contained higher oxygen levels (Fig. S2). More wrinkles were observed on HOGOs because of the higher oxygen levels (Fig. S1). Another GO with a high level of oxygen was prepared through chemical oxidation, similar to the control group.

10 mg of HOGO (GOs-70 h) was dispersed in 2 mL of water, and ultrasonicated for 30 min. Two 5 mg samples of $Pb(NO_3)_2$ were added into the HOGO dispersion (5g/L) at 4 and 50 ℃, respectively. The dispersion was then observed using TEM after a strong shock. The results showed that CGOs could only be formed in the saturated $Pb(NO_3)_2$ or $Co(NO_3)_2$ solution at 4 ℃ (Fig. S6). Even if CGOs were formed, they would still disappear after re-dilution. Meanwhile, the SEM and TEM analysis results showed that no cubic crystals of $Pb(NO_3)_2$ were observed in CGOs, and a large number of ~20 nm spheroids were found (Fig. S3a). These results showed that the CGO formation strongly depended on the temperature and $Pb(NO_3)_2$ concentration. In addition, $Pb(NO_3)_2$ was not detected inside CGOs, showing that CGOs could form in solution under these conditions. Therefore, $Pb(NO_3)_2$ was adsorbed on the outside surface of CGOs after entering the solution, and crystallized on the outside surface, thus fixing the shape of CGOs during the sample preparation for TEM analysis. Fig. S4c shows that CGOs were observed in HOGOs prepared through chemical oxidation, demonstrating that the CGO formation is influenced by the GO oxygen level.

Fig. S6 shows that CGOs were not found in the supersaturated KNO_3 and $NaNO_3$

solutions. Researchers have reported that $Pb(NO_3)_2$ can adsorb onto the GO carboxyl groups to prepare RGO-supported palladium nanoparticles [2]. Therefore, CGO formation could be attributed to the strong $Pb(NO_3)_2$ coordination ability. CGO formation also depended on the adsorption time and the oxygen level. However, Fig. S7 shows that the ultrasonication time could affect the shape of CGOs. We found that $Pb(NO_3)_2$ was wrapped by the GOs (Fig. S7) and evenly distributed in the inside portions of GOs at high radiation doses (Fig. S8). Previous studies have confirmed that more $Pb(NO_3)_2$ would adsorb onto the surface of GOs with higher oxygen levels. This effect causes more $Pb(NO_3)_2$ molecules to be distributed in the inner surface of CGOs, which changes the CGO shape.

In summary, these results showed that GOs with higher oxygen levels could not uniformly disperse in aqueous solutions. As the temperature decreases, GOs would roll into capsules with lower surface energies. To observe the CGO shape through TEM is difficult, and the salting-out of $Pb(NO_3)_2$ could fix the CGO shape such that it could be observed through TEM. However, when CGOs are unstable, CGOs would open again with increasing temperature.

TGA curve of GO-arginine and GO-proline in nitrogen flow

There was an obvious weight loss process in 150-200 ^oC, which is caused by the pyrolysis of oxygen containing functional groups [3,4]. However, the weight loss process caused by nitrogen-containing groups is not obvious [5]. So, the weight process in 150-200 ^oC could be used to represent the content of oxygen groups, and the higher degree of weight loss in 150-200 ^oC shows more oxygen groups and fewer nitrogen groups. Thus, we could use the slope of curves (K) to represent the degree of weight loss. Compared with GO-amino acids, K of GOs is the biggest (K_{A1}=K_{B1}=0.170). The K of GO-D-amino acids (K_{A2}=K_{B2}=0.060) is higher than that of GO-L-amino acids (K_{A2}=0.053, K_{B3}=0.050), which could be due to the higher oxygen content of GO-D-amino acids. The weight loss curve is odd for GO-L- proline. The brief increase of weight could be observed in 450-550 ^oC, which might be due to the adsorption of N_2 or density changes caused by the N_2 flow on GOs. Moreover, the weight loss process of 550-700 ^oC is due to the desorption of L-proline and

sp^2-hybridzied carbon atoms [6].

Preparation of permeation equipment

The filter equipment was successfully designed and prepared according to experimental requirements (Fig. S10). The two MNGM-PET sides have different physical and chemical properties. The PET side is unstable under basic conditions, and the PET membrane may affect the metal ion permeation, such that the metal ions with high pH would be poured into the sink close to the PET side. This sink was referred to as the permeation sink. HCl was used as the driving liquid, and was poured into the sink close to the graphene side.

Selective permeation of differential valence metal ions

KCl, $CaCl_2$, and $FeCl_3$, and NaCl, $MgCl_2$, and $FeCl_3$ (0.1 mol/L per ion, pH 2.5) were, respectively, divided into two groups. The filtration experiments of the two groups were performed using the above methods, and all ions were ranked in a filter sequence. The filter sequence of the metal ions with different valence is $K^+ > Fe^{3+} > Na^+ > Ca^{2+}$, Mg^{2+} (Fig. S11). This sequence could be attributed to the properties of each ion. Fe^{3+} has a higher charge, and is a large hydrated ion. Mg^{2+} and Ca^{2+} are lower down the sequence relative to Na^+, and this result could also be attributed to the lower mobility and larger hydrated ionic radius (Tables S1 and S2).

Effect of pH gradient on permeation

Considering that the filtering capability of K^+ is the strongest among all metal ions, this ion was used to study the effect of the pH gradient on permeation. The results showed that the permeation strongly depended on the pH gradient (Fig. 14), indicating that high H^+ concentrations could enhance metal ion permeation, which is ascribed to ion exchange. The poor performance of the MNGM-PET and PET membranes resulted from the shorter filtration time.

Simulation of the permeation process using the law of Poisewille before 30 s

What pattern could be used to describe the pattern of rapid filtration before 30 s? Supposing that permeation is similar to liquid flow across tubes, and the flow state is coupled with the law of Poisewille, then K^+ transport across the conical hole could be measured through Poisewille formula [7] for liquid laminar flow.

Based on the Poisewille formula given as: $Q = \frac{\pi r^4\ \Delta p}{8\eta L}$, where r is the radius of tube; Δp is difference between the pressures; η is solution viscosity; and L is tube length. As the tube has conical holes, the Q of the flow could be represented as: $Q = \frac{3\pi D^3 d^3 \prod \rho_N A}{128\eta l(D^2+Dd+d^2)}$, where D is the bottom diameter of nanohole; d is the top diameter of the nanohole; Π is the pressure difference between the membranes; A is the effective permeation area of the membrane; η is the solution viscosity; l is the effective membrane thickness; and ρ_N is the number density of holes on the membrane, which could be counted using statistics as $\rho_N = 2.16 \times 10^8$ /cm^2. The results showed that ~9.35 mg/L of K^+ could permeate the MNGM-PET and PET membrane nanoholes, which are close to the measured value (~8.75 mg/L, Fig. S14). These similar values indicated that the metal ion permeation is a laminar flow state, and the permeation power is caused by the permeation pressure. Moreover, ions with high speeds, such as K^+, would mainly distribute in the middle layer of channels under the influence of the microelectric field in the conical tubes. The slow ions, such as Na^+, would mainly distribute in the marginal layer.

Permeation of ions through PET membrane

Several works have reported that the nuclear pore membrane could be used to selectively filter some ions [8-10]. Fig. 6 shows that K^+ and Cs^+ could be limitedly separated through a single PET membrane; however, Fig. S13 shows that ion selectivity was not observed for the metal ions with different valence. Fig. 5c shows that the PET membrane has nanopores with larger scales (small-scale is ~15 nm, large scale is ~50 nm) and an extremely long tube (~20 ìm). The PET tube membrane has a large number of carboxyl groups [11,12], which induce strong interactions with the inner surface of the tube. In fact, this process is utilized in chromatographic columns for separating metal ions, and causes a certain degree of ion selectivity for K^+ and Cs^+. As the adsorption of metal ions onto carboxyl groups increases, the adsorption rapidly reaches equilibrium. Therefore, a certain degree of ion selectivity for K^+ and Cs^+ before 30 s was achieved. However, this selectivity would change after 30 s. As the PET membrane nanopores are large (Fig. 1b), they could not be used as the “filter-tip”

without strong ion exchange properties. The permeation process would be in a state of balance after 30 s, and thus a single PET membrane could not be used to selectively permeate ions.

3. The support figures and tables from references

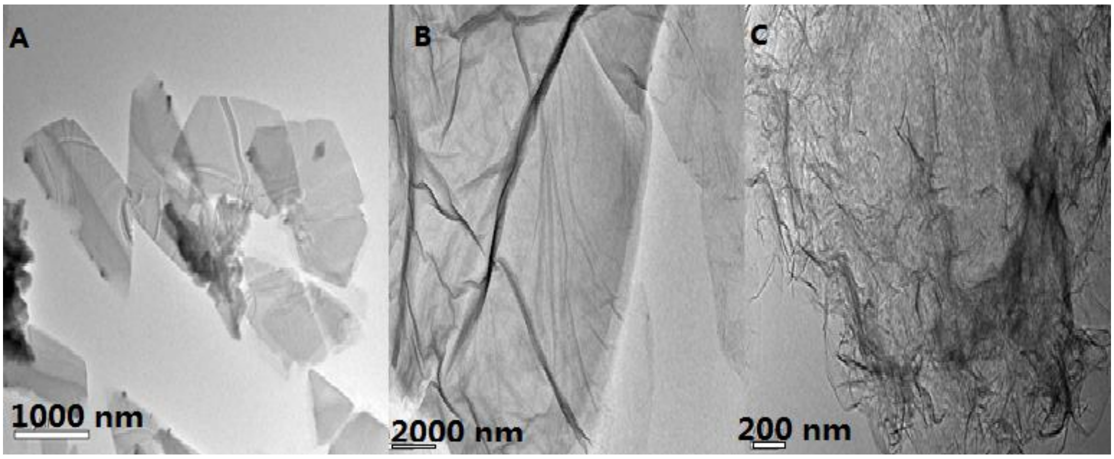

Figure S1. The TEM of graphene **(A)**, common GOs **(B)**, HOGOs **(C)**. The common GO is prepared from graphite oxidated for 18 h; HOGO is the graphene oxide prepared from graphite oxidated for 70 h. Graphene **(A)** is very flat sheets, GO **(B)** sheets exhibit relatively flat surfaces compared with HOGO **(C)**, which is due to the change of oxygen level on GOs.

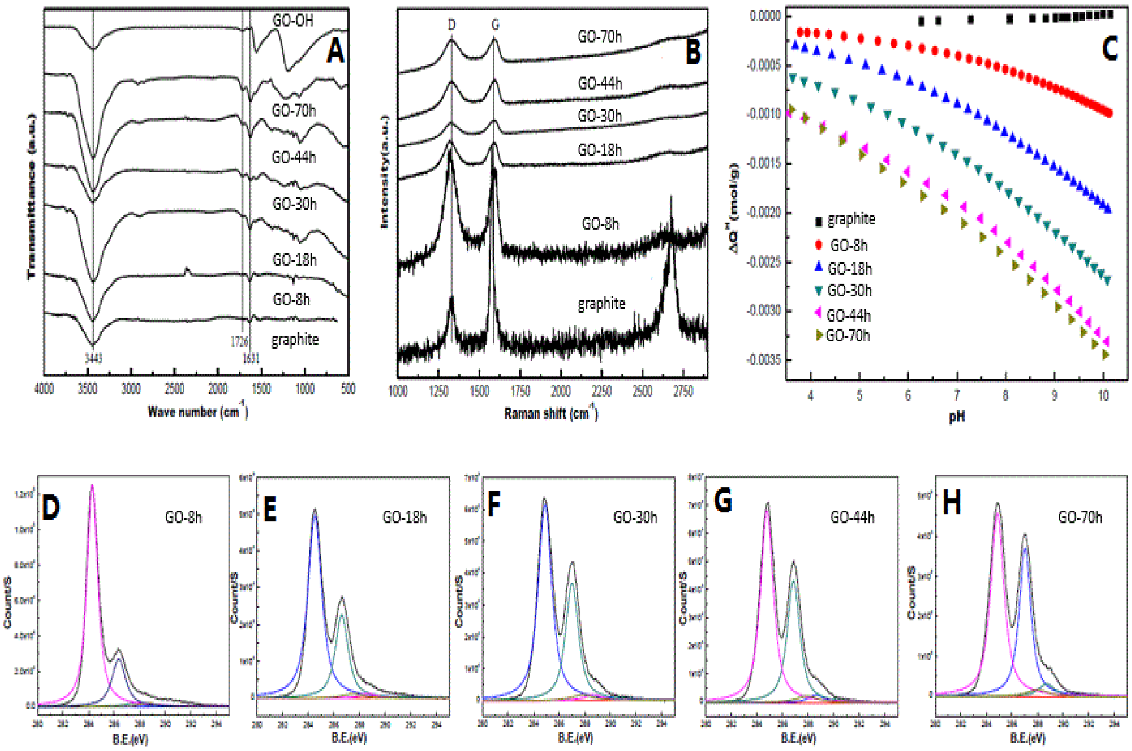

Figure S2. The FTIR **(A)**, Raman spectrum **(B)**, Potentiometric titration curve **(C)** and

XPS **(D-H)** of GOs-8, 18, 30, 44, and 70 h. The time (8, 18, 30, 44, and 70 h) is the oxidation time of graphene prepared from graphite. **(A)** shows that the peak intensity of ~1700 nm in FTIR gradually increased with the increase of oxidation time, and **(B)** shows that the band of *D*2 (~ 2625 cm^{-1}) in Raman Spectrum in GO with longer oxidation time (> 18 h) is lower than common graphite and GOs-8 h, the ~1700 nm is peak of –COOH, the *D*2 band represents the number of layers of graphene, so GOs with longer oxidation time contain higher oxygen level and lesser number of layers. **(C)** shows that GOs with longer oxidation time exhibit obvious effects of deprotonation with the increase of pH, which is due to the increase of oxygen level (-COOH and –OH) on GOs. **(D-H)** also confirm that oxygen double bond (~ 246 eV) on GOs increased with increase of oxidation time.

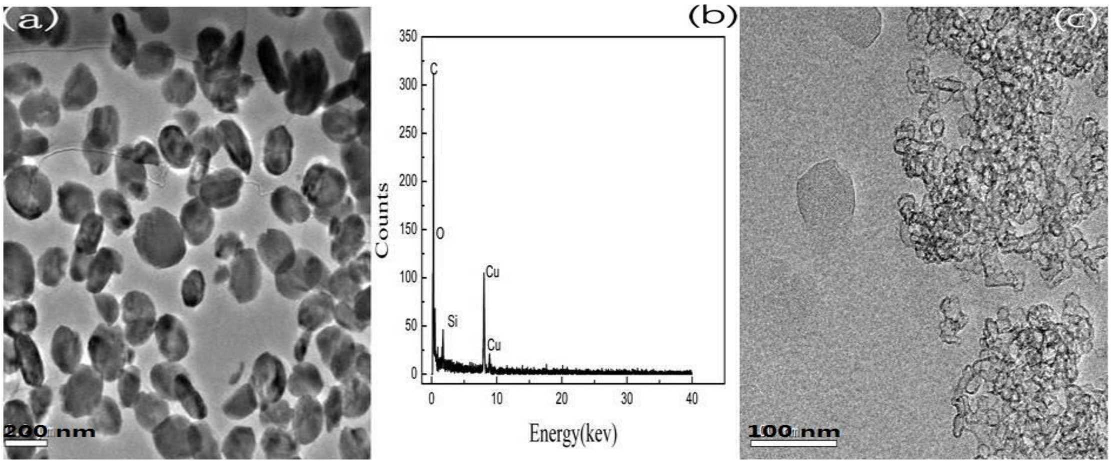

Figure S3. The TEM and EDS of CGOs.

(A) CGOs were from HOGOs. **(B)**, the EDS of CGOs in (A), the Cu and Si in **(B)** came from supporting film of samples. **(C)**, the CGOs was formed from the high oxygen level GOs using normal chemical oxidation.

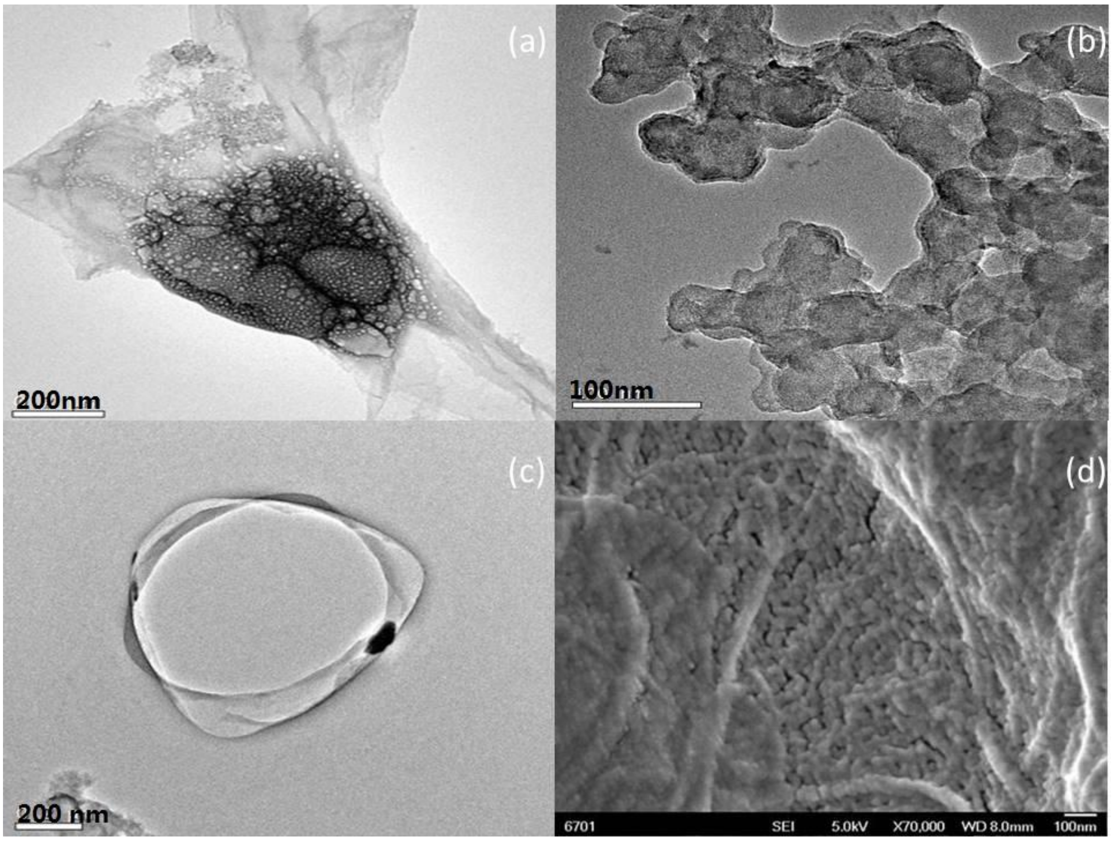

Figure S4. The effect of temperature on the formation of CGOs.

(A), the TEM of products in 50 ^{o}C. **(B)**, the TEM of multilayer CGOs in 4 ^{o}C. **(C)**, the TEM of monolayer CGOs in 4 ^{o}C. **(D)**, the SEM of CGOs in 4 ^{o}C in supersaturation $Pb(NO_3)_2$.

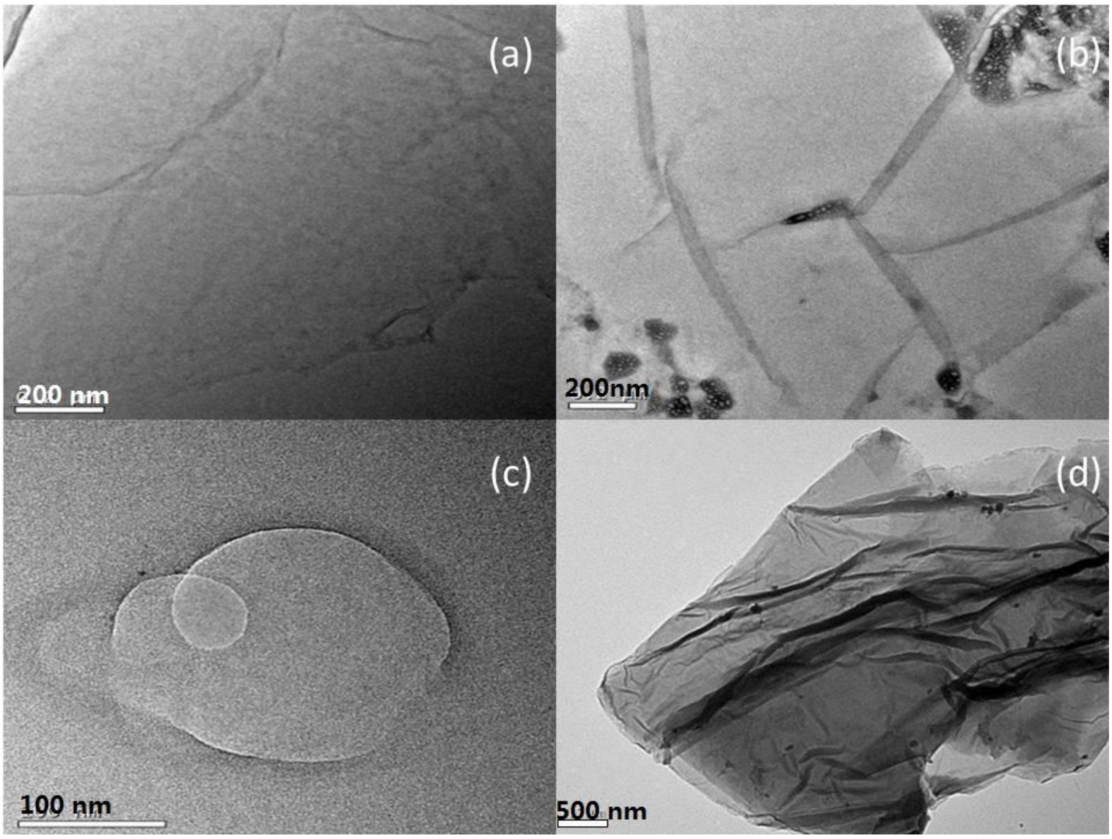

Figure S5. The effect of salt concentration on the CGOs in 4 ^{o}C.

(A), 0.12 mol/L $Pb(NO_3)_2$. **(B)**,1.51mol/L $Pb(NO_3)_2$. **(C)**, 2 mol/L $Pb(NO_3)_2$. **(D)**, the re-dilution of CGOs.

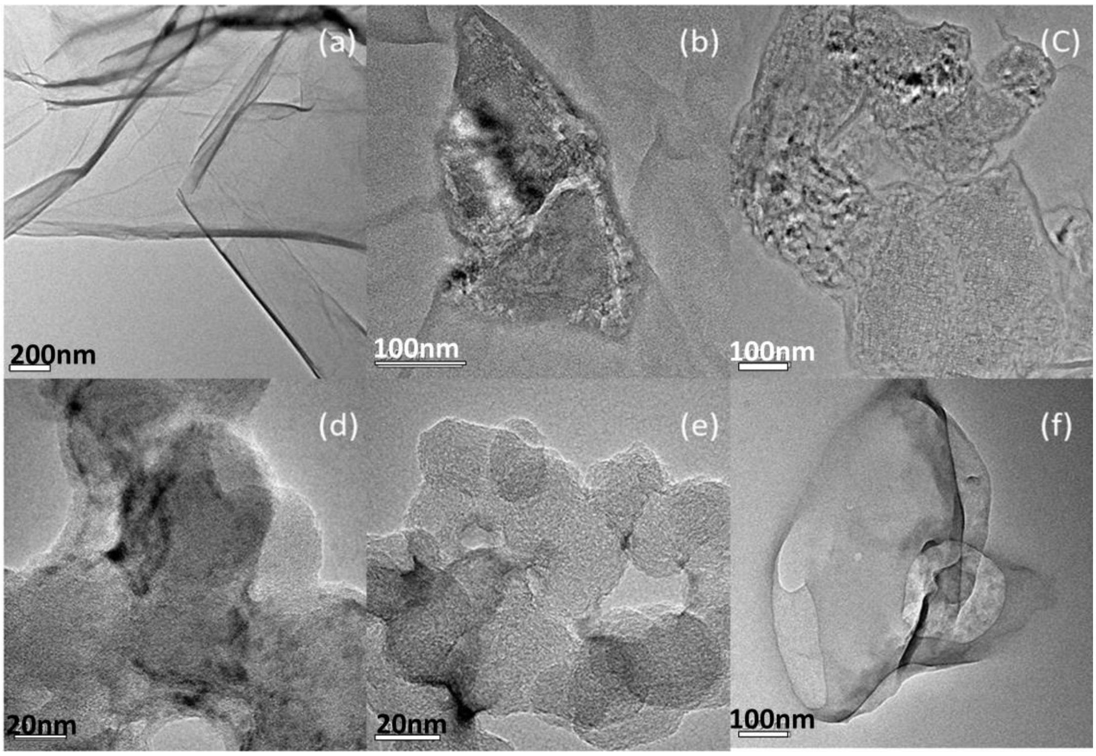

Figure S6. The formation of CGOs in different salt saturated solution in 4°C. **(A)**, free salts. **(B)**, KNO_3. **(C)**, $NaNO_3$. **(D)**, $Co(NO_3)_2$. **(E)**, multilayer CGOs in $Pb(NO_3)_2$. **(F)**, monolayer CGOs in $Pb(NO_3)_2$.

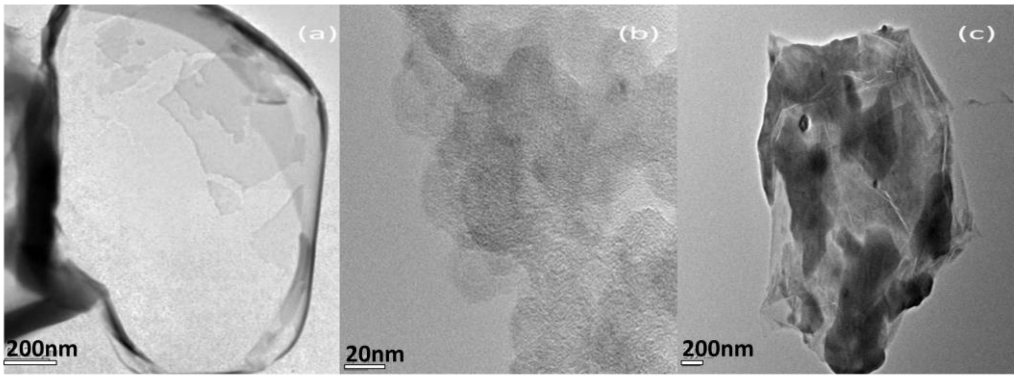

Figure S7. The effect of ultrasonic time on the formation of CGOs in 4 ^{o}C. **(A)**, 0 h (monolayer). **(B)**, 0 h (multilayer). **(C)**, 24 h (monolayer).

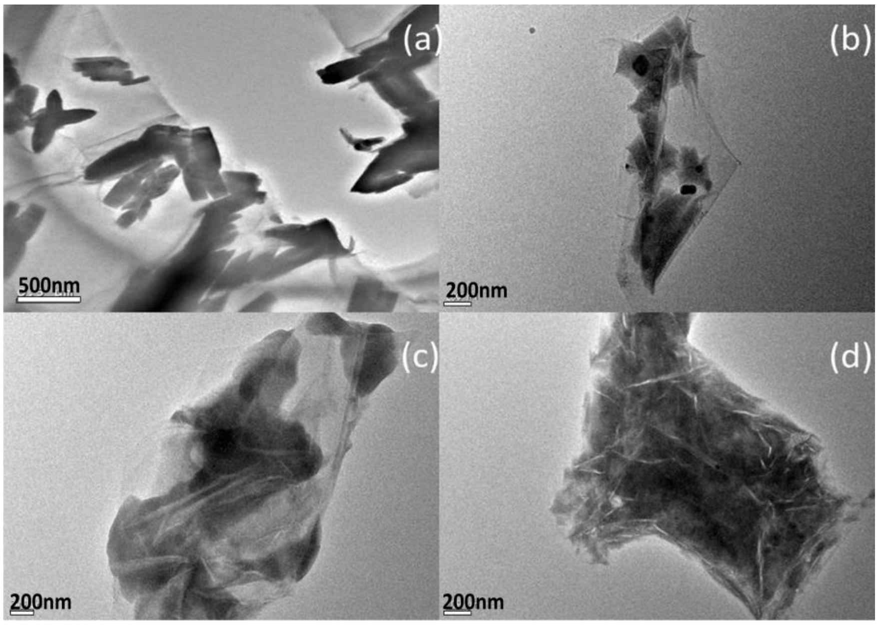

Figure S8. The effect of oxidation time on the formation of CGOs after ultrasound for 4h in 4°C. (A), 24 h. **(B)**, 48 h. **(C),** 70 h. **(D)**, 96 h.

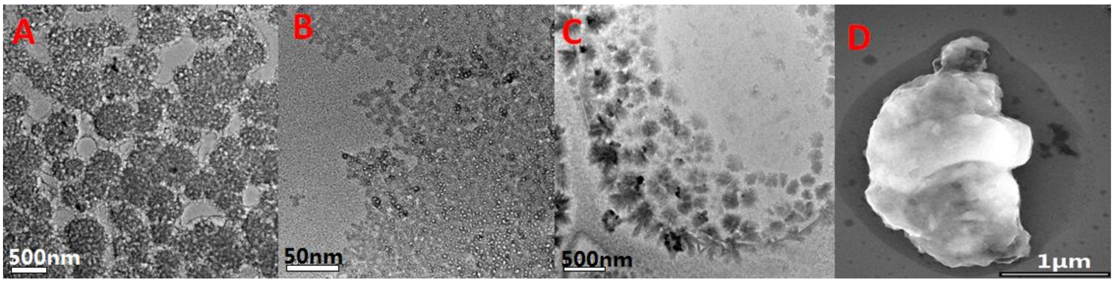

Figure S9. The TEM of DOPC+$Pb(NO_3)_2$ and GOs+DOPC+$Pb(NO_3)_2$. **(A)**, composite of DOPC and $Pb(NO_3)_2$ without GOs. **(B)**, composite of DOPC and $Pb(NO_3)_2$ in the presence of GOs. **(C)**, the TEM of CGO with DOPC, the burr-like materials is the composite of DOPC and Pb^{2+}. **(D)**, the SEM of CGO with DOPC, the white particle is crystals of $Pb(NO_3)_2$, a layer of black graphene wrapped around salt.

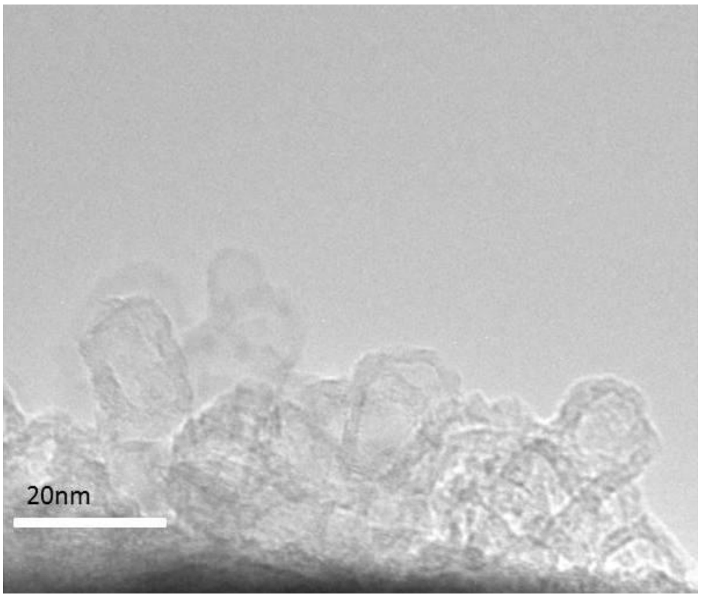

Figure S10. The CGOs formed after reaction between HOGOs and amino acid. The HOGOs- glycine in reaction products could roll into the CGOs at 4 ^{o}C in the saturated $Pb(NO_3)_2$ solution.

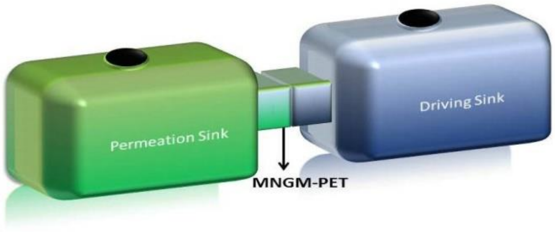

Figure S11. The permeation installations of metal ions. The HCl as a driving liquid

was poured into the driving sink, the salt solution as a permeation liquid was poured into the permeation sink.

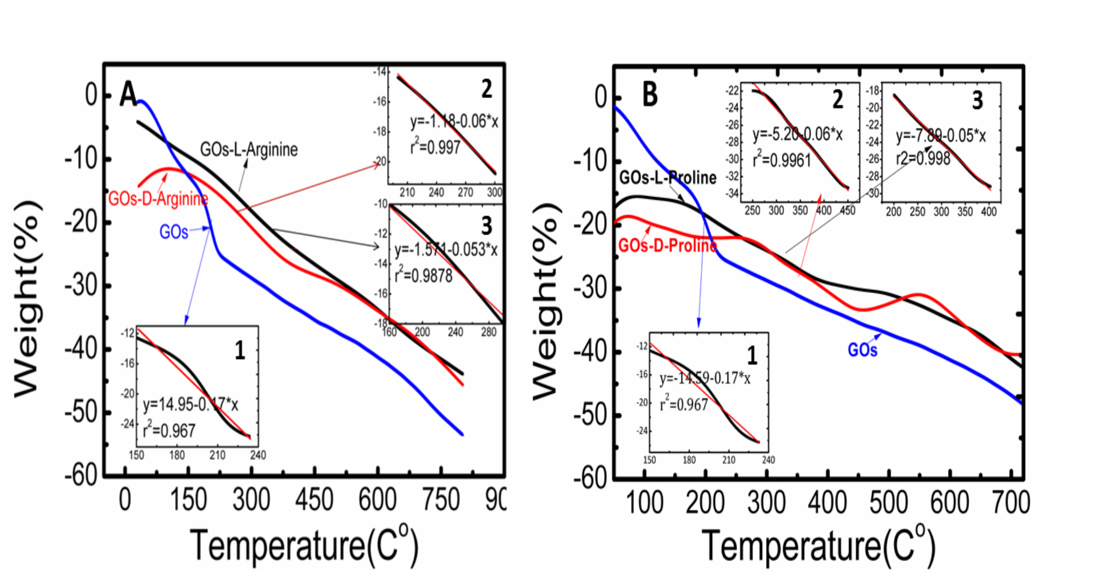

Figure S12. The TGA of GO-amino acids under nitrogen. (**A**), the GOs-arginine. (**B**), the GOs–proline. The (A/B-1,-2 and -3) is the curve (black) and fitting linear (red) of weightlessness of –COOH/OH. $A_{1,\ 2}$ and $_3$ is GOs, GO-L-Arginine, and GO-D-Arginine, respectively; $B_{1,\ 2}$ and $_3$ is GOs, GO-L-Proline, and GO-D-Proline, respectively. The slope of linear fit represents the degree of weightlessness. K_{A1}=0.170>K_{A2}=0.060>K_{A2}=0.053; K_{B1}=0.170>K_{B2}=0.060>K_{B3}=0.05.

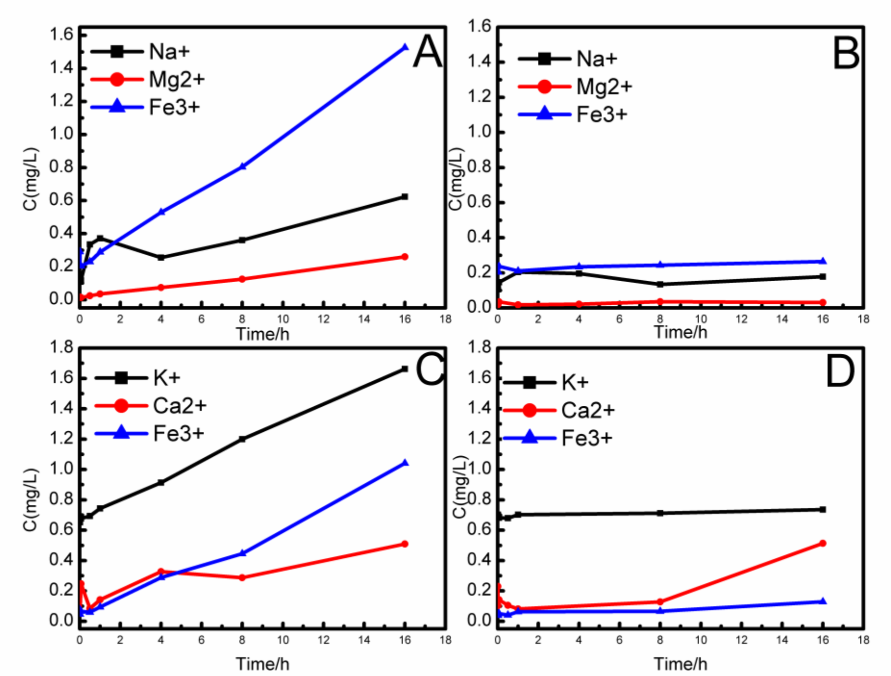

Figure S13. The selective filter of MNGM-PET and PET membrane on different

valence metal ions. ΔpH=1.5, T=25°C, C_{ions}=0.1mol/L. The **(A)**, **(C)** are the filter of MNGM-PET; the **(B)**, **(D)** are the filter of PET.

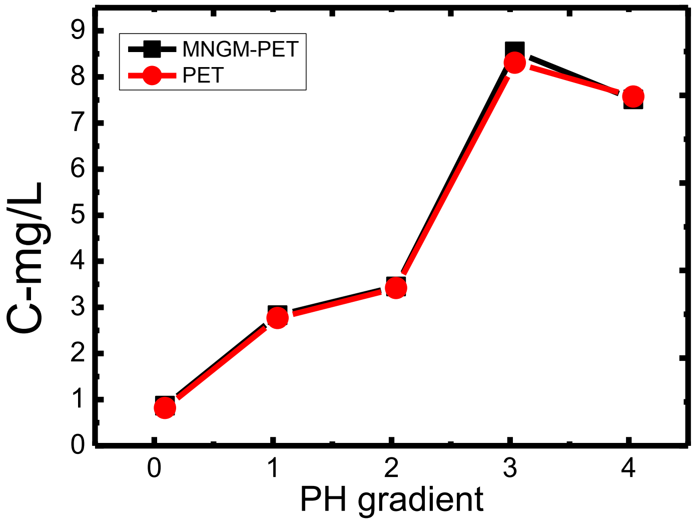

Figure S14. The effect of pH gradient on the permeation of K^+. The filtration time is 1 h, the diving liquid is HCl.

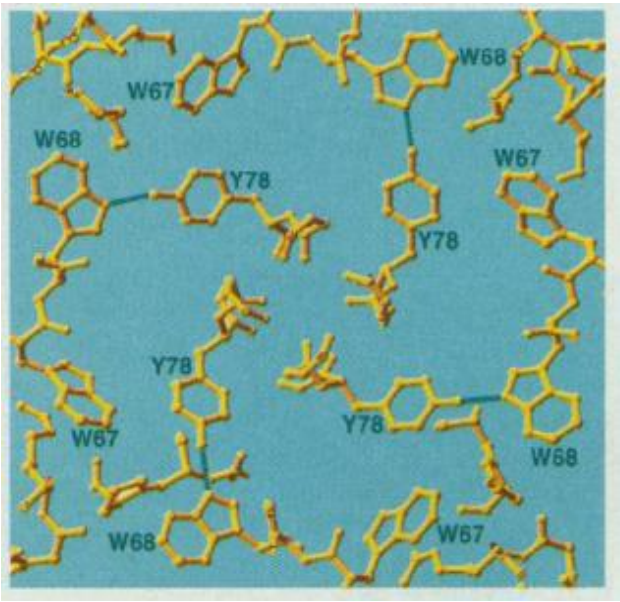

Figure S15. A section of the model perpendicular to the pore at the level of the selectivity filter and viewed from the cytoplasm [13].

The view highlights the network of aromatic ammo acids surrounding the selectivity filter. Tyroslne-78 from the selectivity filter (Y78) interacts through hydrogen bonding and van der Waals contacts with two Trp (W67,W68) from the pore helix.

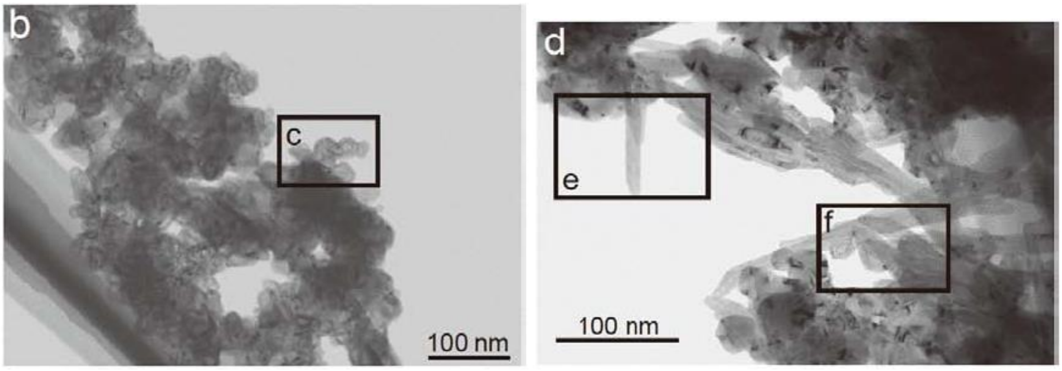

Figure S16. The TEM of Ohtomo about early life before 3.7 billion years ago. (Ohtomo, Nature Geoscience, 2014) [14]. **(b)**, Cummingtonite, graphite, and quartz are the dominant minerals in sample 4062308. **(d)**, TEM image of graphite (bright field image) in sample 6072906.

Table S1. The ion mobility of different metal ions [15]

Ions	Li^+	Na^+	K^+	Rb^+	Cs+	Mg^{2+}	Ca^{2+}
Ion mobility [$U_+ \times 10^8 (m^2 s^{-1} V^{-1)}$]	4.01	5.19	7.62	8.46	9.63	5.50	5.90

Table S2. The hydrated ionic radius of different ions [15]

Ions	Li^+	Na^+	K^+	Rb^+	Cs^+	Fe^{3+}	Mg^{2+}	Ca^{2+}
Hydrated ionic radius(Å)	3.82	3.58	3.31	3.29	3.29	4.57	4.28	4.12

References

(1) Hummers, W. S.; Offeman, R. E. *J Am Chem Soc* **1958**, *80*, 1339.

(2) Yang, S.; Dong, J.; Yao, Z.; Shen, C.; Shi, X.; Tian, Y.; Lin, S.; Zhang, X. *Sci. Rep.* **2014**, *4*, 4501.

(3) Stankovich, S.; Dikin, D. A.; Piner, R. D.; Kohlhaas, K. A.; Kleinhammes, A.; Jia, Y.; Wu, Y.; Nguyen, S. T.; Ruoff, R. S. *Carbon* **2007**, *45*, 1558.

(4) Fan, Z. J.; Kai, W.; Yan, J.; Wei, T.; Zhi, L. J.; Feng, J.; Ren, Y. M.; Song, L. P.; Wei, F. *ACS Nano* **2011**, *5*, 191.

(5) Hu, Y.; Shen, J.; Li, N.; Shi, M.; Ma, H.; Yan, B.; Wang, W.; Huang, W.; Ye, M. *Polymer Composites* **2010**, *31*, 1987.

(6) Mei, X.; Ouyang, J. *Carbon* **2011**, *49*, 5389.

(7) Pfitzner, J. *Anaesthesia* **1976**, *31*, 273.

(8) Ovchinnikov, V. V.; Seleznev, V. D.; Surguchev, V. V.; Tokmantsev, V. I. *J Membrane Sci* **1991**, *55*, 311.

(9) Happel, S.; Streng, R.; Vater, P.; Ensinger, W. *Radiat Meas* **2003**, *36*, 761.

(10) Guo, S.-L. *New Astronomy Reviews* **1998**, *42*, 205.

(11) Singh, N. L.; Singh, D.; Qureshi, A. *Radiat Eff Defect S* **2011**, *166*, 640.

(12) Kumar, V.; Sonkawade, R. G.; Chakarvarti, S. K.; Singh, P.; Dhaliwal, A. S. *Radiat Phys Chem* **2012**, *81*, 652.

(13) Doyle, D. A.; Cabral, J. M.; Pfuetzner, R. A.; Kuo, A. L.; Gulbis, J. M.; Cohen, S. L.; Chait, B. T.; MacKinnon, R. *Science* **1998**, *280*, 69.

(14) Ohtomo, Y.; Kakegawa, T.; Ishida, A.; Nagase, T.; Rosing, M. T. *Nature Geosci* **2014**, *7*, 25.

(15) Veazey, W. R.; Hodgman, C. D.; Chemical Rubber Company.; Chemical Rubber Pub. Co.: Cleveland,, p 57 volumes.
